# Second-Generation Sequencing Supply an Effective Way to Screen RNAi Targets in Large Scale for Potential Application in Pest Insect Control

**DOI:** 10.1371/journal.pone.0018644

**Published:** 2011-04-11

**Authors:** Yubing Wang, Hao Zhang, Haichao Li, Xuexia Miao

**Affiliations:** Key Laboratory of Insect Developmental and Evolutionary Biology, Institute of Plant Physiology and Ecology, Shanghai Institutes for Biological Sciences, Chinese Academy of Sciences, Shanghai, People's Republic of China; AgroParisTech, France

## Abstract

The key of RNAi approach success for potential insect pest control is mainly dependent on careful target selection and a convenient delivery system. We adopted second-generation sequencing technology to screen RNAi targets. Illumina's RNA-seq and digital gene expression tag profile (DGE-tag) technologies were used to screen optimal RNAi targets from *Ostrinia furnalalis*. Total 14690 stage specific genes were obtained which can be considered as potential targets, and 47 were confirmed by qRT-PCR. Ten larval stage specific expression genes were selected for RNAi test. When 50 ng/µl dsRNAs of the genes DS10 and DS28 were directly sprayed on the newly hatched larvae which placed on the filter paper, the larval mortalities were around 40∼50%, while the dsRNAs of ten genes were sprayed on the larvae along with artificial diet, the mortalities reached 73% to 100% at 5 d after treatment. The qRT-PCR analysis verified the correlation between larval mortality and the down-regulation of the target gene expression. Topically applied fluorescent dsRNA confirmed that dsRNA did penetrate the body wall and circulate in the body cavity. It seems likely that the combination of DGE-tag with RNA-seq is a rapid, high-throughput, cost less and an easy way to select the candidate target genes for RNAi. More importantly, it demonstrated that dsRNAs are able to penetrate the integument and cause larval developmental stunt and/or death in a lepidopteron insect. This finding largely broadens the target selection for RNAi from just gut-specific genes to the targets in whole insects and may lead to new strategies for designing RNAi-based technology against insect damage.

## Introduction

RNA interference (RNAi) refers to double-stranded RNA (dsRNA) mediated gene silencing [1]. Since its discovery, it has been widely used in insect genetic research [2], [3], [4]. Recently, a new hot point is to find a feasible way to use RNAi as an alternative method for practical application of crop protection [5], [6], [7]. In this aspect, two major technologies are considered as the most important, namely, a simple delivery system and a high throughput screen method of optimal RNAi target genes, especially in those pests lacking any genome or transcriptome sequence information.

The dsRNA is delivered either by injection or by feeding insects. It has been reported that in several insect orders the gene can be knocked down by dsRNA injection [8], [9], [10], [11], but injection is not applicable to control pest insects in the field. Oral delivery of dsRNA was demonstrated in the nematode *Caenorhabditis elegans* [12], [13]. Since then, it was reported in a few insect species that dsRNA can be integrated in artificial diets and results in target gene knockdown [14], [15]. In addition, two research papers indicate that transgenic crops can produce dsRNAs to protect the plant against insect feeding damage [16], [17]. However, this technique is relatively complex and premature in terms of practical application in pest insect control. Thus, a more convenient way should be explored before dsRNA can be used as insecticides.

Regarding the optimal target gene selection, taking advantage of the known genes and cDNA library screening were the main ways up to now [14], [15], [17], [18]. Herein, the selection of target genes from the considered pest insects is still a major challenge. A high throughput screening system of RNAi target genes will be more appreciated when RNAi-based pest insect control can be taken in consideration. Recent results suggest that the next-generation sequencing (NGS) technologies allow directly sequencing the cDNA generated from messenger RNA (RNA-seq) [19], [20]. This new method for analyzing RNA-seq data enables the *de novo* reconstruction of the transcriptome for a nonmodel organism [21]. NGS technologies have led to novel opportunities for expression profiling in organisms lacking any genome or transcriptome sequence information [22], [23]. Can these technologies be used in RNAi target genes screening?

Asian corn borer *Ostrinia furnalalis* (Guenée) (ACB) is a major pest of corn in eastern and south-eastern Asia. At present, its control relies mainly on insecticides. Although the transgenic crops producing *Bacillus thuringiensis* (Bt) toxins for the pest insect control have been succeeful, their efficacy was gradually reduced when pests evolved resistance [24], [25], [26]. Thus there is an urgent need to find an alternative solution to control pest insects. The RNAi-based technology may be considered as an ideal alternate measure when efficient systems of dsRNA formulation and delivery have been developed [5], [7].

In this study we report that RNAi-induced lethality could be achieved by direct delivery via the exoskeleton; moreover an effective high-throughput NGS method was used to select stage-specific RNAi target genes in the Asian corn borer.

## Results

### Transcriptome of the ACB by RNA-seq

To obtain a global view of transcriptom in the whole ACB life cycle, high-throughput RNA-seq was performed by Solexa/Illumina sequencing technology in Beijing Genomics Institute at Shenzhen. We obtained more than 33 million paired-end reads of 75 bp in length. The total lengths of the reads were over 2.5 giganbases (Gb). The clean reads were *de novo* assembled into 124,034 contigs with the assemble software of SOAP*denovo* [27] (Table 1). Then all the reads were realigned onto contigs again according to the paired-end reads overlap relationship, and all these contigs were joined into 79,825 scaffold sequences. Finally, the intra-scaffold gaps were filled using the paired-end extracted reads. Those sequences which cannot extend to any direction were called unigene. We obtained 45,750 unigenes for the ACB in this project (Table 2). The unigenes were annotated with the database of Nr, Swiss-Prot, KEGG, COG and Gene Ontology (GO) function analysis. The residual unigenes were predicated using the software of ESTscan [28].

**Table 1:** The contigs assemble results of *O. furnacalis* transcriptome.

	100∼200 nt	200∼300 nt	300∼400 nt	400∼500 nt	≥500 nt	N50*	Mean	Total	Length of all contig (nt)
Contig number	88,670	17,490	8,431	3995	5,457	211	198	124,043	24,499,499
Percent (%)	71.48	14.10	6.80	3.22	4.40				

*N50 size of contigs was calculated by ordering all sequences then adding the lengths from longest to shortest until the summed length exceeded 50% of the total length of all sequences.

**Table 2:** The scaffold and unigene assemble result of *O. furnacalis* transcriptome.

	100∼500 nt	500∼1000 nt	1000∼1500 nt	1500∼2000 nt	≥2000 nt	N50*	Mean	Total	Total length (nt)
Scaffold number	65,965	10,308	2,403	716	433	455	334	79,825	26,641,821
Percent (%)	82.64	12.91	3.01	0.90	0.54				
Unigene number	31,899	10,302	2,398	719	432	555	485	45,750	22,205,852
Percent (%)	69.72	22.52	5.24	1.57	0.94				

*N50 size of contigs was calculated by ordering all sequences then adding the lengths from longest to shortest until the summed length exceeded 50% of the total length of all sequences.

### The analysis and statistics of DGE-tag sequences

**Figure 1:**
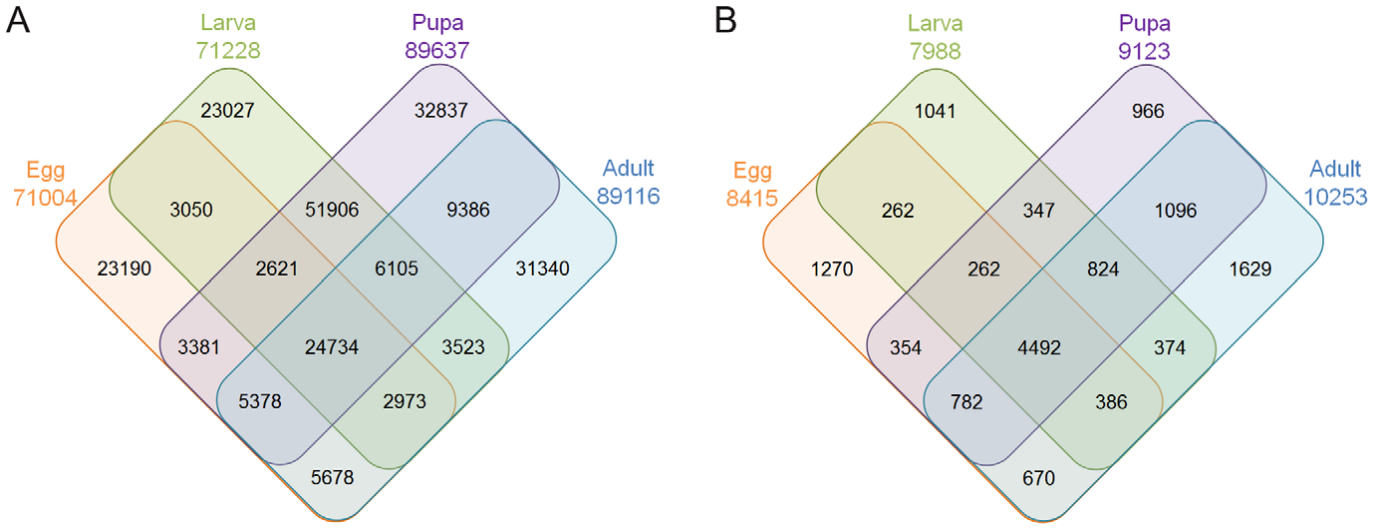
Venn diagram showing unique and shared gene expression among the ACB. (**A**) All clean tag differential expression and co-expression between 2 to 4 developmental stages in four developmental stages (Egg, Larva, Pupa and Adult); (**B**) These tag sequences were mapping by RNA-seq dataset. We obtained 8415, 7988, 9123 and 10253 annotation unigenes for egg, larvae, pupal and adult stages, respectively. Among all of these unigenes, the differential expression and co-expression clean tags and unigenes between 2 to 4 developmental stages were shown.

We sequenced four DGE-tag libraries from four ACB developmental stages of egg, larva, pupa and adult. After eliminating low quality tags (containing Ns), copy numbers less than 2 and adaptor sequences, the numbers of total clean tags were about 3.57, 3.54, 3.55 and 3.45 millions for eggs, larvae, pupae and adults, respectively. The four successive developmental stages possessed 71004, 71228, 89637 and 89116 clean tag sequences, respectively. These tag sequences were composed of the DGE-tag datasets presented in Table 3. When the clean tags were annotated with the ACB RNA-seq dataset, 8415, 7988, 9123 and 10253 annotation unigenes were obtained for eggs, larvae, pupae and adults, respectively. Among them, 14690 stage specific unigene tags are unambiguous and can be considered as potential targets (Table S1). Among all of these unigenes, the differential expression and co-expression of clean tags and unigenes between 2 to 4 developmental stages are shown in Figure 1A and 1B.

**Table 3:** DGE-tag raw data and clean tag amount in the four developmental stages of *O. furnacalis.*

	Egg		Larva		Pupa		Adult	
	Total Tags	Unique Tags	Total Tags	Unique Tags	Total Tags	Unique Tags	Total Tags	Unique Tags
Raw Data	3,695,998	190,977	3,663,435	191,840	3,704,416	236,104	3,601,481	230,618
Low quality	14,412	7,036	14,207	6,990	14,701	8,028	14,177	8,094
Adaptors	0	0	0	0	0	0	0	0
CopyNum<2	112,937	112,937	113,622	113,622	138,439	138,439	133,408	133,408
Clean Tag	3,568,649	71,004	3,535,606	71,228	3,551,276	89,637	3,453,896	89,116

To obtain the profile of the differential expression genes, the distribution of tag copy numbers in each of the four stages, is summarized in Table 4.

**Table 4:** The distribution of clean tag copy number in the four developmental stages of *O. furnacalis*.

Tag copies	Egg	Larva	Pupa	Adult	Total
2	22,429	22,416	28,418	28,979	102,242
3∼9	28,164	28,837	36,678	35,670	129,349
10∼49	13,682	13,802	17,167	16,720	61,371
50∼99	2,908	2,646	3,341	3,515	12,410
100∼199	1,860	1,594	1,957	2,027	7,438
200∼299	664	597	635	728	2,624
300∼399	318	269	369	367	1,323
400∼499	205	182	219	241	847
500∼999	383	397	446	442	1,668
1000∼1999	212	238	193	236	879
2000∼4999	101	158	146	133	538
5000∼9999	39	59	42	35	175
≥10000	39	33	26	23	121
Total	71,004	71,228	89,637	89,116	320,985

### Analysis of 10 larval stage specific genes and qRT-PCR

**Figure 2:**
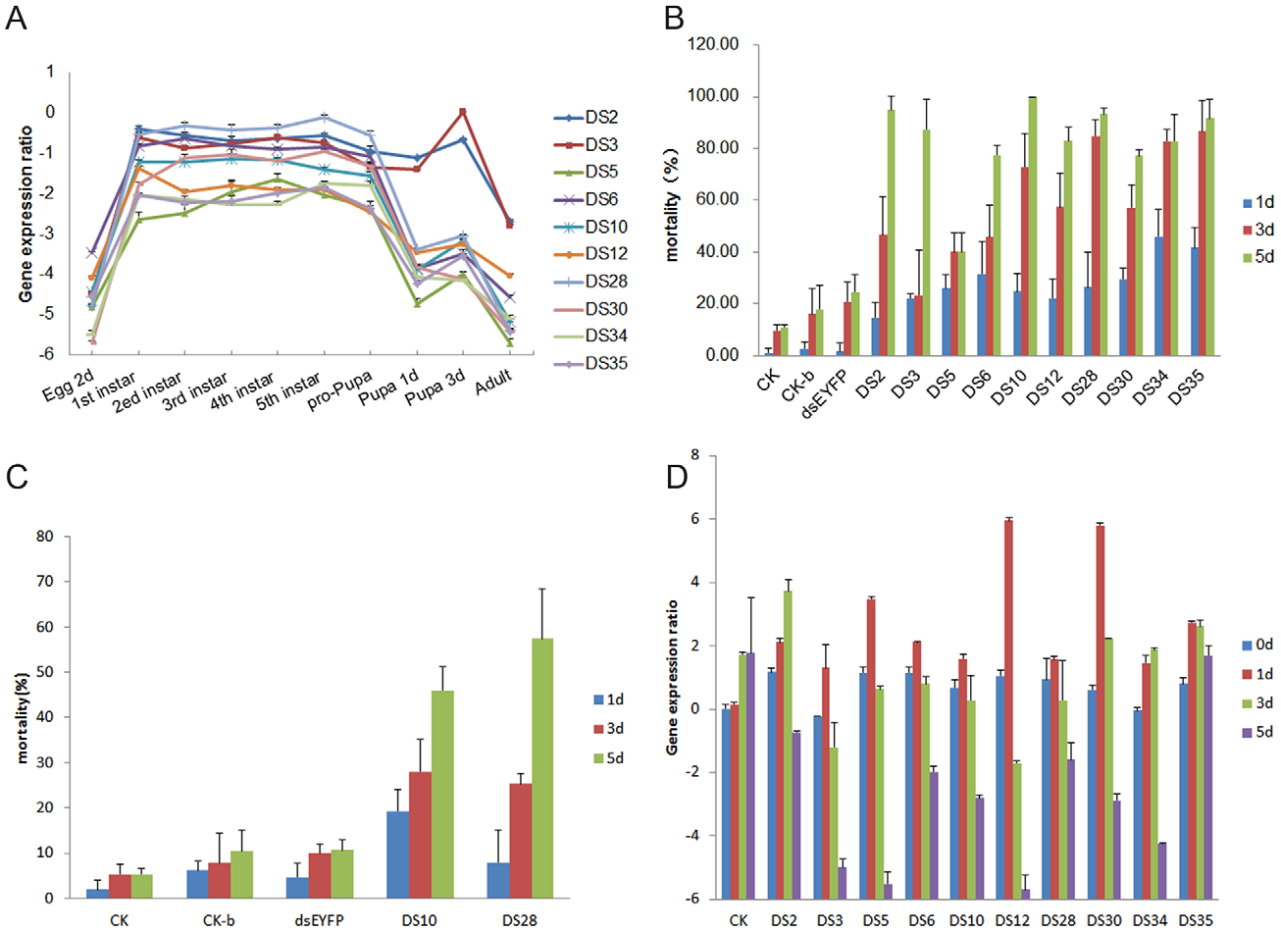
The relative expression level of 10 larval stage specific genes and the larval mortality. (**A**) The relative expression levels of 10 genes by qRT-PCR. All 10 genes were showing larval stage specific expression genes, and each of them was selected according to the DGE-tag copy number (**Table 5**). The expression profile was examined from egg to adult stage in which the larval stage involved 1^st^ to 5^th^ instar larvae and pre-pupa. The results were consistent with their DGE-tag copy numbers as shown in **Table 5**, indicating all of them were larval stage specific except DS2 and DS3 which had relatively higher expression level at pupal and adult stages. It was also consistent with the tag copy number (**Table 5**). To normalize threshold cycle (*C_t_*) values obtained for each gene, 18S rRNA expression levels were used. (**B**) The larval mortality of dsRNA directly sprayed on the larvae along with artificial diet. Ten dsRNAs, 50 ng/µl for each (**Table 5**) were directly sprayed on the newly hatched larvae on artificial diet respectively. dsEYFP and the blank buffer were used as controls. The larval survival was monitored in a 5 d period. At day 5 the corrected mortality reached to more than 70% except DS5. (The data were also shown in **Table 6**). (**C**) The larval mortality of dsRNA directly sprayed on the larvae which were placed on filter paper. 50 ng/µl dsRNA of DS10 and DS28 were directly sprayed on the newly hatched larvae on a filter paper pre-soaked in the buffer, and then the larvae were transferred to artificial diet. The blank buffer and dsEYFP were used as controls. The larval survival was monitored in a 5 d period. (**D**) The 10 gene expression levels at different time after dsRNA treatment. The expression level of 10 genes increased at 1 d after dsRNA spraying (the red bars). 8 out of the 10 genes began to decrease at 3 d after treatment except DS2 and DS34 (the green bars). However, the expression levels of all 10 genes decreased in different degree 5 d after treatment (the purple bars).

At first, we selected 47 larval stage high expression tags to determine their expression levels by qRT-PCR. All of them indeed appear consistent with their DGE-tag copy numbers (Table S2). To screen target genes used for producing RNAi of ACB, 10 larval genes with higher expression levels were selected according to the DGE-tag copy number. The cDNA sequences of these tags and their predicted functions were acquired from the RNA-seq dataset and Genebank (Table 5). The qRT-PCR analysis confirmed that the relative expression levels of all 10 genes in the larval stage were higher than those in other developmental stages except DS2 and DS3 (Figure 2A). Results are consistent with their tag copy numbers as seen in Table 5.

**Table 5:** Ten larval stage specific expression gene tag sequences, copy number in each stage and their function prediction.

Name	GI	Tag sequences	Egg	Larva	Pupa	Adult	Gene function
DS2	GH989961	GACGCTGGTGAACACCT	0	6,325	1,942	1,136	LIM protein 1(*Lonomia oblique*)
DS3	GH993407	CTGACCGCCGCCGCGGA	0	5,134	2,839	550	myosin 3 light chain(*Lonomia oblique*)
DS5	GH998766	GTTTAACAGCCATATGT	0	3,281	0	0	chymotrypsin-like serine protease(*Ostrinia nubilalis*)
DS6	EL929307	GGGCTTCCCCTGCGCCC	8	2,244	0	8	chymotrypsin-like protease C1(*Heliothis virescens*)
DS10	GH999144	CACCCTCAGTGGACCCC	0	1,239	2	0	chymotrypsin-like serine proteinase C3(*Ostrinia nubilalis*)
DS12	GH989348	GTGTTCGCGTGAACAAC	15	1,229	29	64	hydroxybutyrate dehydrogenase(*Heliothis virescens*)
DS28	GH997930	AGAATAATGCAAGCTTG	0	566	0	0	unknown(*Helicoverpa armigera*)
DS30	GH989017	ACGAGCTATCATCGCCT	0	537	0	0	Kazal-type serine proteinase inhibitor 1(*Bombyx mori*)
DS34	EL928502	CAGTACAAGCCGAACCA	0	421	2	0	Fatty acid-binding protein 1(*Mamestra configurata*)
DS35	GH994832	ATGGTTCTTTATCCAAC	4	390	0	0	caboxypeptidase 4(*Mamestra configurata*)

**Table 6:** The eggs hatch ratio and larval mortality (%) after spraying 50 ng/µl dsRNA.

Name	Egg hatch ratio	1d mortality	3d mortality	5d mortality	5d-CK* 5d-EYFP*	
CK	97.15±2.89	1.00±1.73	9.67±2.31	11.00±1.00		
CK-b	80.68±7.28	2.67±2.52	10.50±3.54	13.00±4.24		
dsEYFP	75.26±11.37	5.33±1.15	10.00±2.00	14.67±5.03		
DS2	18.41±11.18	14.67±5.86	46.6±14.74	95.00±5.20	94.38	94.14
DS3	58.94±18.89	22.00±2.00	23.3±17.24	87.33±11.72	85.77	85.16
DS5	49.97±5.81	26.00±5.29	40.00±7.21	40.00±7.21	32.58	29.68
DS6	57.61±18.89	31.6±12.52	45.7±12.31	77.42±3.83	74.63	73.54
DS10	60.67±19.14	24.86±6.68	72.9±12.89	100.00±0.00	100.00	100.00
DS12	43.92±2.13	22.08±7.61	57.3±13.18	83.08±5.03	80.99	80.17
DS28	49.72±8.11	26.3±13.35	84.86±6.41	93.29±2.51	92.46	92.13
DS30	41.04±19.63	29.59±4.11	57.21±8.68	77.27±2.29	74.46	73.36
DS34	36.68±10.78	46.0±10.63	82.71±4.70	82.87±10.41	80.76	79.93
DS35	40.30±14.09	41.66±7.77	86.7±11.72	91.55±7.62	90.51	90.10

CK is the control of feeding on artificial diet;

CK-b is the control after spraying with dsRNA blank buffer;

dsEYFP is the negative control of the treatment with dsRNA of exogenous EYFP gene;

*5 day corrected mortality by CK and dsEYFP.

### RNAi in ACB via a direct spray of dsRNA

To explore a convenient way of dsRNA delivery, 10 *in vitro* synthesized dsRNAs (Table 5) were sprayed uniformly on the newly hatched larvae along with an artificial diet. The blank buffer, dsEYFP-containing buffer and artificial diet were used as controls. When 50 ng/µl was sprayed, 9 out of 10 dsRNAs caused a significant developmental stunting and a high level of mortality. After correction, the 5 day mortalities levels were between 73% and 100%. Actually dsRNAs targeting DS2, DS10, DS28 and DS35 led to more than 90% death (Figure 2B; Table 6). As in this experiment dsRNAs were sprayed on the larvae along with artificial diet, there might be two possible pathways of dsRNA delivery, either by penetration of the body wall or by feeding. To test the first possibility (through the body wall), we sprayed 50 ng/µl dsRNAs targeting DS10 and DS28 to the newly hatched larvae placed on moist filter paper and then the larvae were transferred to artificial diet. As a result, 5 day corrected mortalities levels of 40% and 52% for DS10 and DS28 were obtained respectively (Figure 2C), LC_50_ were 61.02 (53.67–69.14, P<0.05) ng/µl for DS10 and 60.44 ng/µl (51.84–87.13, P<0.05) for DS28. This indicates that dsRNA can be delivered into haemolymph through the exoskeleton and still causes insect mortality. Another evidence is produced using labeled fluorescent dsRNA (see below).

To investigate the correlation of larval mortality with the target gene expression, the expression levels of all 10 genes were measured by qRT-PCR after spraying 50 ng/µl dsRNA. The expression levels of the 10 genes increased 1 day after dsRNA treatment (Figure 2D, red bar). Three days later, profiles began to decrease except that of DS2 and DS34 which showed a slight increase (Figure 2D, green bar). All gene expression levels were knocked down to a certain degree 5 days after dsRNA treatment (Figure 2D, purple bar).

**Figure 3:**
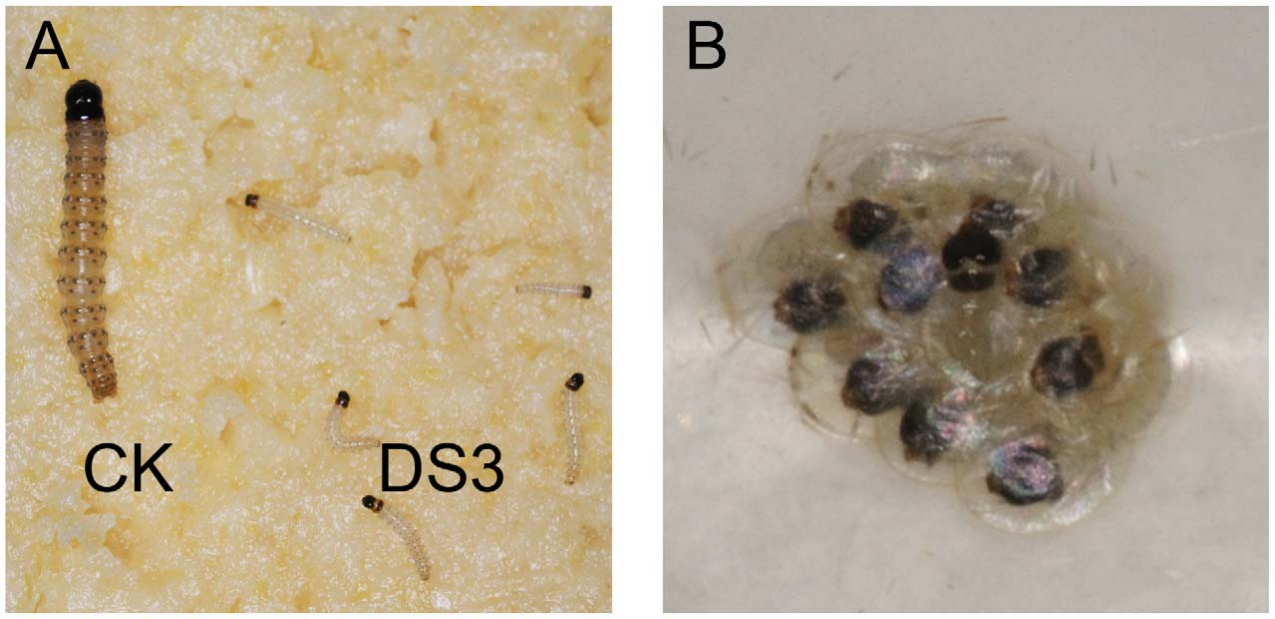
The eggs were soaked in dsRNA solutions or the dsRNA was sprayed on larvae. (**A**) When 20 ng/µl dsRNA (DS3) feeding solution was sprayed on the larvae, the development of the treated larvae was stunted remarkably. CK (left side): one of the control larvae reaching third instar 7 d after spray with dsRNA blank buffer, DS3 (right side): the larvae remaining in first instar 7 d after spraying dsRNA of DS3 gene; (**B**) The egg masses were soaked in 50 ng/µl dsRNA solution for 2h, the embryos could develop, but most of them were not able to hatch.

When 20 ng/µl dsRNA solution was sprayed on the larvae along with the artificial diet, the larval development was remained in the first instar until 7 days after treatment and a certain degree of larval death was seen when the control larvae had reached the third instar stage (Figure 3A). When ACB eggs were soaked in 50 ng/µl dsRNA solution for 2 hours, all 10 genes caused an obvious decrease of hatching ratio compared with controls, although results varied for different genes (Table 6; Figure 3B).

### Use of fluorescent labeled dsRNA

**Figure 4:**
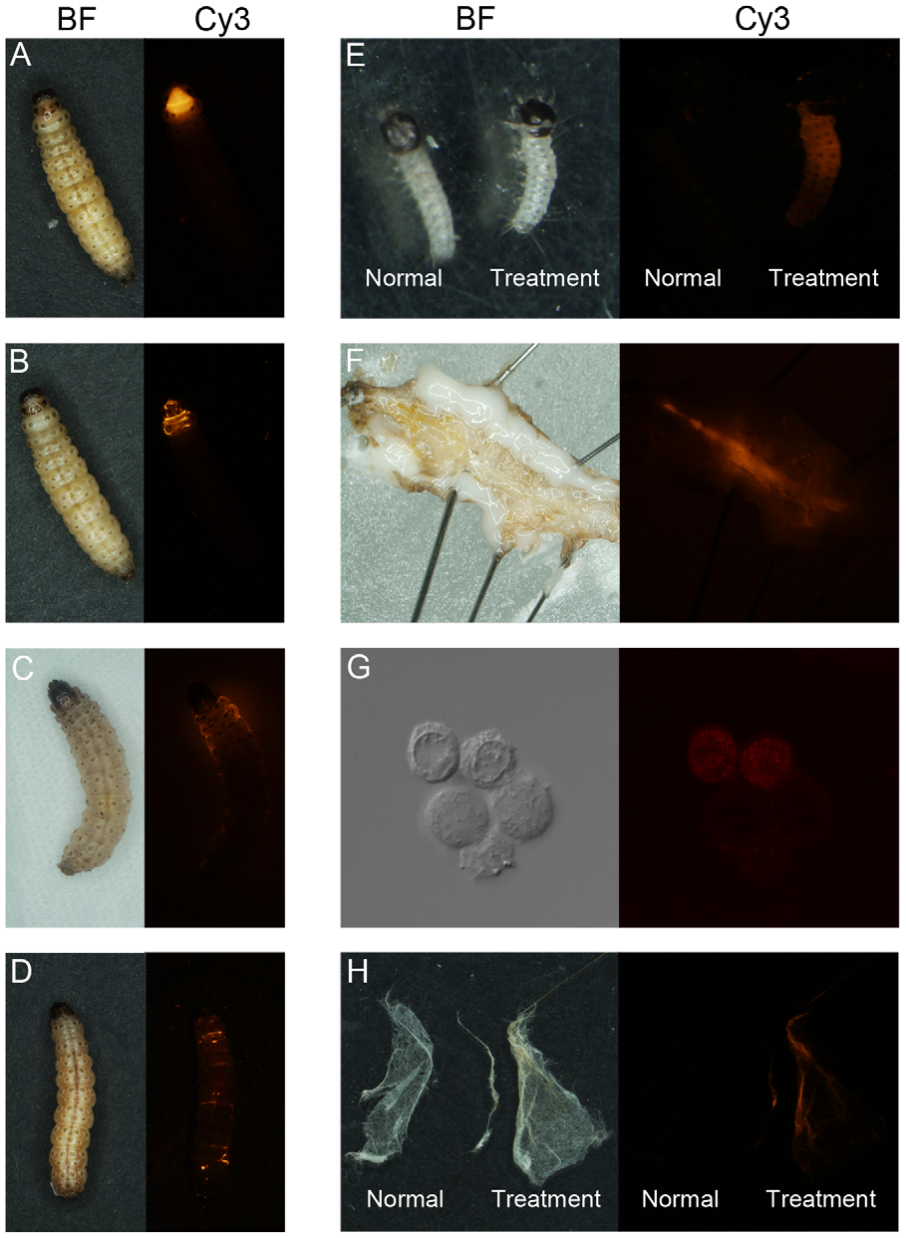
The fluorescent labeled dsRNA dropping test. The fluorescent labeled dsRNA (0.5 µl of 5 µg/µl) was topically applied on the protergum of the ACB mature larva. (**A**) The droplet was seen at the surface of the body wall just after dropping; (**B**) 30 min later, the droplet had already penetrated the body wall, and spread to the head and other thoracic segments; (**C**) 2 h later, the fluorescent dsRNA had spread to abdominal segments; (**D**) 4 h later, the fluorescence was seen in the whole body; (**E**) When the egg was soaked in the solution with 50 ng/µl fluorescent labeled dsRNA for 2 h, the newly hatched larvae were labeled with fluorescent; The fluorescence was observed in the gut(**F**), hemocytes (**G**) and silk fiber (**H**). From **A** to **H**, the left panel of each pair was on bright field (BF) and the right panel of each pair was on 559nm (Cy3). Image magnification: **A–D**, **F**, **H** (10×); **E** (40×); **G** (400×).

To further confirm that dsRNA can penetrate the egg shell and the larval body wall of ACB, a fluorescent nucleotide was added during the dsRNA synthesizing of gene DS12. When 0.5 µl of 5 µg/µl fluorescent labeled dsRNA was topically applied on the larval protergum of the matured last instar larvae, fluorescence showed that penetration through the larval cuticle took only a few minutes and in 4 hours the whole body was reached (Figure 4, A–D). After 2 hours soaking the eggs in the fluorescent labeled dsRNA solution in a concentration of 50 ng/µl, the fluorescence was observed in the newly hatched larvae (Figure 4E). The gut (Figure 4F), hemocytes (Figure 4G) and silk fiber (Figure 4H) were also clearly labeled, suggesting that the delivered dsRNA not only penetrated into the body cavity but also circulated in heamolymph. To avoid the possibility of the droplet placed on the prothorax moved to the head and taken up orally by the larvae, we dropped 0.5 µl of 5 µg/µl fluorescent labeled dsRNA on the fifth abdominal tergum of the mature larvae, and a similar pattern was seen (figures not shown).

## Discussion

Taking advantage of known genes is a simple and effective way to verify RNAi targets, but the scope of selection was limited [15], . cDNA library screening approach can identify targets that would not necessarily be predicted from functional consideration but has the drawback of being rather labour-intensive comsuming if a large number of insect bioassays are required [14], [17]. RNA-seq is a recently developed deep-sequencing technology. After comparison with the existing technologies (DNA microarrays, cDNA or EST sequencing), Wang *et al.* (2009) summarized the advantages of RNA-seq [19]. This sequencing-based method does not include any cloning or amplification step, and requires less RNA sample. Furthermore, RNA-seq is not limited to detecting transcripts corresponding to existing genomic sequences, thus it is especially suitable for non-model organisms [19], [20]. The 3′-tag digital gene expression (DGE-tag) is also a second-generation sequencing-based technology. Asmann *et al.* (2009) comparing DGE-tag with the microarray technology, emphasized that this method has a high sensitivity and reproducibility for transcriptome profiling [23]. Therefore, we chose this method to screen the target genes for RNAi test in this study. Using this method, among the 14690 stage specific unigene tags, 10 larval stage specific genes were tested, 9 of them were confirmed as effective RNAi targets. It indicated that the combination of DGE-tag with RNA-seq is a rapid, cost less and easy way to select candidate target genes for RNAi in comparison with cDNA library screening.

Successful and unsuccessful RNAi experiments have been reviewed in a number of lepidopteran species by Terenius *et al*. (2011). As indicated in this review, the majority of RNAi studies in Lpidoptera were done using *Bombyx mori*, *Manduca sexta* and some Noctuidae species. Systemic RNAi has been demonstrated in some species, such as *Hyalophora cecropia* and *B. mori*, in which injection of dsRNA into the pupa can result in phenotypic effects in developing embryos, indicating dsRNA uptake by the developing oocytes of the pupa. However, a great variation of sensitivity to systemic RNAi has been seen among different lepidopteran species and high or no silencing can occur at very different concentrations of dsRNA [31]. Therefore, more work using different species and alternative delivery protocols seems helpful. It is generally thought that either injection or feeding are feasible ways to deliver dsRNA to produce a RNAi effect and potentially cause the development delay and death in insects [6], [18], [29]. Our results revealed that a direct spray of dsRNA can produce such effect in the ACB larvae, including the down-regulation of dsRNA targeted gene expression, developmental delay and death (Figures 2B and 2C; Table 6). In one experiment, after spraying DS10 and DS28 dsRNA on the newly hatched larvae placed on moist filter paper, their development was stunted and they finally died after 5 days of treatment. Corrected mortalities were 40% for DS10 and 52% for DS28. This indicates that the sprayed dsRNAs did penetrate the exoskeleton and could reach hemolymph and produce RNAi effect. In the previous reports no one had tried this kind of method for crop pest insects as it was believed that dsRNA could only go through insect midgut, the only region not covered by chitin exoskeleton. Huvenne and Smagghe deduced that sufficiently protected dsRNA, e.g. by some sort of coating, should be used to spray [7]. Results of the topically application of the fluorescent labeled DS12 dsRNA (Figure 4) further demonstrated that dsRNA does penetrate the integument, enters into heamolymph and labeled several tissues such as midgut, haemocytes and silk fiber. DS12 encoding protein was identified as a lipid requiring mitochondrial enzyme [32]. This suggests that the introduced dsRNA may circulate in heamolymph of ACB larvae. We still do not know whether the dsRNA is able to directly penetrate the body wall from the integumental membrane or reach the site of action via the integument of the tracheal system. However, this finding provides new insights that RNAi targets can be selected from the whole insect instead of gut-specific targets after feeding with dsRNA. So far, only one report had provided evidence that topically applied *AaeIAP1* dsRNA products in acetone dilution was able to kill adult female mosquito *Aedes aegypti* [33]. In our experiments, two dsRNAs were directly spraying on larvae placed on filter paper. They match the genes encoding chymotrypsin-like serine protease C3 (DS10) and an unknown protein (DS28) (Table 5). Chymotrypsin-like serine protease was reported to be involved in food digestion in some lepidopteron insects [34] and interacted with the chitin synthesis from the midgut of *Maduca sexta* [35]. Recently a trypsin-like protease gene has been cloned in the integument of *Helicopverpa armigera* [36]. Thus, it is possible that this family gene encoding proteins may also interact with the chitin synthesis of the cuticle in dsRNA treated ACB larvae. In another experiment, we sprayed 10 larval stage specific genes dsRNA on the newly hatched larvae along with artificial diet. As a result, 9 of them caused a significantly developmental stunting and 5 days corrected mortalities reached high levels (between 73% and 100%) (Figure 2B; Table 5). And even four dsRNAs resulted in more than 90% mortality; matching genes of DS2, DS10, DS28 and DS35, which encode LIM protein, chymotrypsin-like serine protease C3, unknown protein and caboxypeptidase 4. The partial function of DS35 and DS10 is associated with the digestion in the midgut [37], [38], but LIM protein (DS2) is localized in muscle and was suggested to act as adapters capable of mediating different protein-protein interactions [39], [40]. DS35 was detected in the molting fluid possibly involved in the degradation of proteins from the old cuticle in *B. mori* [41]. It indicates that the observed RNAi effects can be achieved through a variety of targets in insects. The qRT-PCR analysis verified the correlation between larval mortality and the down-regulation of the target gene expression (Figure 2C). It is reasonable to think that dsRNA delivery through both the integument contact and oral ingestion may enhance RNAi effect leading to larval death. Because nine of the ten genes tested had a measureable impact on larval mortality, in order to exclude non-specific effects, we tested two more ACB genes, namely a pupal specific gene encoding the transcription elongation factor B polypeptide 3 binding protein (P18) and an adult gene encoding the hypothetical protein (A4). They show high expression levels only in pupae (P18) and adults (A4), respectively, but no expression in other stages. After dsRNAs of these two genes (50 ng/µl) were sprayed on the newly hatched larvae along with artificial diet, the larval mortality caused was similar to that of DS5, the only less susceptible gene among ten genes tested in terms of the RNAi induced larval death (data not shown). It suggests that dsRNAs of the higher expressed larval genes may achieve more effective gene silencing and higher mortality of ACB larvae.

In conclusion, our results demonstrate that a direct delivery via integument is a feasible way to introduce dsRNA into the haemolymph, which then leads to a down-regulation of gene expressions and finally to the development retarding and/or death. However, some questions still remain to be further investigated, such as in which tissues or cells these genes are silenced and whether the proposed receptor mediated endocytosis or the transmembrane channel-mediated uptake are the mechanisms leading to the production of RNAi effect as well as the pathway of RNAi effect leading to larval developmental stunt and death in ACB larvae. Nevertheless, our findings considerably broaden the selection of targets for RNAi research from gut-specific to whole insects. It may lead to new strategies in designing the RNAi-based technology against insect damage.

## Materials and Methods

### Insect culture

Asian corn borer *Ostrinia furnacalis* (Guenée) eggs were obtained from Sun Yat-Sen University and reared in laboratory at 25°C and 75% relative humidity on a 14-h-day/10-h-night cycle. Larvae were fed on a modified artificial diet (maize granule 120.0 g, maize flour 32.0 g, soybean flour 120.0 g, Vc 4.0 g, agar-agar 12.0 g, yeast powder 72.0 g, sorbic acid 4.0 g, glucose 60.0 g, gormaldehyde 1.6 mL, water 1000 ml).

### Sample collection and RNA isolation

24 samples were collected from egg to adult stages during the whole life cycle of the ACB. The samples were immediately frozen in liquid nitrogen, and stored at −80°C before RNA extraction. Total RNA was isolated using a Qiagen RNA Extraction kit according to the manufacturer's instructions. It was treated with RNase-free DNase I for 30 min at 37°C (New England BioLabs) to remove residual DNA. Then the equivalent amount of the 24 samples was merged into 4 pools of egg, larva, pupa and adult. The total RNA in the whole life cycle came from the 4 pools in equivalent amounts. mRNA was isolated from DNA-free total RNA using Dynabeads mRNA Purification Kit (Invitrogen).

### cDNA synthesis, sequencing and data analysis of RNA-seq

First-strand cDNA was synthesized using Oligo(dT)_18_ primer and reverse transcriptase (Invitrogen). The second-strand cDNA was synthesized using RNase H (Invitrogen) and DNA polymerase I (New England BioLabs). Then the 100–500 bp paired-end cDNA library was constructed for sequencing using the Illumina/Solexa technology.

Short read sequences were *de novo* assembled using SOAPdenovo program [27]. The unigene expression level was calculated by RPKM (Reads Per kb per Million reads) method [42]. Short clean reads were loaded into computer and the unambiguous sequence fragments as contigs were output. Then the reads were realigned onto the contigs according to the paired-end information and join the unique contigs into scaffolds. Finally, the intrascaffold gaps were filled using the paired-end extracted reads. Those sequences which can not extend to any direction were unigenes. The unigenes were compared with the database of Nr, Swiss-Prot, KEGG and COG and Gene Ontology (GO) function analysis.

### Sequence tag preparation, sequencing and DGE-tag annotation

Sequence tag was prepared with Illumina's Digital Gene Expression Tag Profiling Kit according to the manufacturer's protocol. For the readers who want to have a schematic overview of the procedure, please see the reference [43]. When the DGE-tag library has been constructed, it was sequenced by Illumina Cluster Station and Genome Analyzer.

We filtered out low quality tags (containing Ns), copy number below 2 and adaptor sequences. At last, we obtained 3.4∼3.5 million clean sequence DGE-tags for each developmental stages of egg, larva, pupa and adult. The DGE tags, which consist of the CATG restriction enzyme digested site and an additional 17 bp from each transcript, were aligned onto the transcriptome unigenes of ACB. The expression abundance of transcripts was measured by the number of tags mapped.

### dsRNA synthesis

dsRNAs were synthesized using the MEGAscript RNAi kit (Ambion, Huntingdon, UK) according to manufacturer's instructions. T7 promoter sequences were tailed to each 5′ end of DNA template by PCR amplifications. Double stranded EYFP (dsEYFP) was generated using pPigbacA3EYFP as template and was used as a negative control in the experiments. All the primer sequences were listed in Table 7. Template DNA and Single-stranded RNA were removed from the transcription reaction by DNase and RNase treatment. dsRNA was purified using MEGAclearTM columns (Ambion) and eluted in 100 ul nuclease free water. dsRNA concentrations were measured using biophotometer (Eppendorf).

**Table 7:** The *in vitro* dsRNA synthesize with T7 tail primers of 10 larval stage specific genes.

NO.	Forward primer	Reserve primer
EYFP	TAATACGACTCACTATAGGGAGGACGACGGCAACTACAAG	TAATACGACTCACTATAGGGGAACTCCAGCAGGACCATGT
DS2	TAATACGACTCACTATAGGGAGAGCACGAGGGCAGTCTCGGC	TAATACGACTCACTATAGGGAGAGCGACCCGCTAGCTCTGCTG
DS3	TAATACGACTCACTATAGGGAGACGCACCGGCTCCAACGTCTT	TAATACGACTCACTATAGGGAGACTGGCGCGGGTGGAACTACG
DS5	TAATACGACTCACTATAGGGAGACTGCGGCAGTTCGCTGGTCA	TAATACGACTCACTATAGGGAGATGGACGCGATGACGATGCCG
DS6	TAATACGACTCACTATAGGGAGAGTCCAGAGCCGCTGGTTGGG	TAATACGACTCACTATAGGGAGACAAGCAACGAAGTGGCGGGC
DS10	TAATACGACTCACTATAGGGAGAGCGTCACCGCTCAGTCCCAC	TAATACGACTCACTATAGGGAGACCGGCAGCAGCACCAAAGGA
DS12	TAATACGACTCACTATAGGGAGACGCTGGAGAGGGAGCGAACG	TAATACGACTCACTATAGGGAGACCTCACCGGCCCAGGGCTAA
DS28	TAATACGACTCACTATAGGGAGAGCAGCTGGCCAAACGACGTG	TAATACGACTCACTATAGGGAGACGGCGCTGTCTTGGACTGGG
DS30	TAATACGACTCACTATAGGGAGAGACCGGACCATGCAGAGGCG	TAATACGACTCACTATAGGGAGAGCACACTTATCAACACGGACAGGG
DS34	TAATACGACTCACTATAGGGAGAGCCTTTGACGCCAACGCCAAG	TAATACGACTCACTATAGGGAGATCTGTAGGCAACGCCATCCCA
DS35	TAATACGACTCACTATAGGGAGACAGCCCCGCCAACTCCTTCG	TAATACGACTCACTATAGGGAGAACGGTCTGGAGTGCAAATGCGT

### Fluorescent labeled dsRNA and the dropping test

The dsRNA synthesis procedure was the same as described above except that 0.25 µl 10 mM fluorescent dCTP labeled with Cy3 was added. Taking advantage of character, T7 RNA polymerase can take dNTP as substrates for synthesis RNA [44]. Then 0.5 µl of 5 µg/µl dsRNA solution was topically applied on the protergum or the fifth abdominal tergum of the ACB mature larvae that had stopped feeding and did not move. The larvae dripped with Cy3 labeled dsRNA were observed under common SZX16 fluorescent microscopy (Olympus) every 0.5 h or 1 h. The haemocytes were observed under confocal fluorescent microscopy FV1000 (Olympus).

### Insect bioassay

Three hundred newly hatched larvae were placed in a culture dish (9 cm in diameter). 300 µl 50 ng/µl dsRNA in dsRNA buffer [14] of different genes was sprayed uniformly on them using the micro-sprayer. The experiments were performed in triplicate. After air dried the treated insects for 15 min, the 300 larvae were equally divided into 3 groups and placed in Petri dishes with artificial diet, then the dish was sealed with Mylar film (Dupont) that was pricked with small holes to provide some aeration without desiccating the food. The survival larvae were counted every day for each treatment. LC_50_ was calculated by using 5 different concentrations, 20, 40, 60, 80, 100 ng/ul dsRNA. The data were analyzed using one-way ANOVA to look for treatment effects compared to the untreated control (P<0.05). In addition, the egg masses were soaked in 50 ng/µl dsRNA soaking buffer for 2 hours and the effect on embryo development and the hatching rate was examined [45].

### Quantitative real-time PCR (qRT-PCR)

Expression levels of 10 larval stage specific genes selected according to the DGE-tag copy number were evaluated by qRT-PCR. Primer sequences were provided in Table 8. A qRT-PCR assay for multiple genes was performed with the SYBR® Premix Ex Taq ™ II (TaKaRa). 18S rRNA expression levels were used to normalize *C_t_* values obtained for each gene. qRT-PCR was carried out using a Mastercycler ep realplex instrument (Eppendorf). Data analysis methods were described as the reference [46].

**Table 8:** The qRT-PCR primers of 10 larval stage specific genes.

NO.	Forward primer	Reserve primer
DS2	CCAAGGGCTACGGCTTCGGC	GCTGCTCGCGCTCAATTCGC
DS3	CGCACCGGCTCCAACGTCTT	GCCGAGCGAGTCGAAGGTGG
DS5	CGTCAGCGGCGGTACCCATC	TGCTGACCAGCGAACTGCCG
DS6	TCGACTTCAACGTCGCCGCC	GCCGGAGTCACCGTTGCACA
DS10	ACCCGTGTGATCACCGCTGC	GGTCTGACGGACACCACCGC
DS12	CGCTGGAGAGGGAGCGAACG	TCGGCGGTGATCACAAGAGGC
DS28	AAAGGCATTCGGGGCCGTCG	TTGGCCAGCTGCCCGCTAAC
DS30	GACCGGACCATGCAGAGGCG	CGGTTGCCGTTGCCGTCACA
DS34	AAGACCCCCGCCCTGAACCA	GCCAAGGGGGCCGTAGTCCT
DS35	CAGCCCCGCCAACTCCTTCG	CCCTGGAGTGGATGCCTCCGT

To assess the extent of RNAi, RNA was extracted from pools of 20∼30 dsRNA-treated and surviving larvae using a Qiagen RNA Extraction kit. Primer sequences were provided in Table 8.The samples were then treated with DNAse I (Invitrogen) to remove any genomic DNA contamination, and were used with Superscript II reverse transcriptase (Invitrogen) to make first strand cDNA using random primers. qRT-PCR reactions and statistic were the same with above.

## Supporting Information

Table S1 **DGE-tag mapping by RNA-seq dataset and annotated.**
(XLS)

Table S2 **Larval stage high expression tags copy number confirmation by qRT-PCR.**
(XLS)
